# Over-supplement of vitamin D may cause delirium, abdominal distension, and muscle weakness in the elderly: A case report and literature review

**DOI:** 10.1097/MD.0000000000041057

**Published:** 2024-12-27

**Authors:** Ching-Hsiang Yu, Hsiang-Kuang Tseng, Ding-Kuo Chien, En-Chih Liao

**Affiliations:** a Department of Emergency Medicine, MacKay Memorial Hospital, Taipei, Taiwan; b Department of Medicine, MacKay Medical College, New Taipei City, Taiwan; c Division of Geriatric Medicine, MacKay Memorial Hospital, Taipei, Taiwan; d Institute of Long-term Care, MacKay Medical College, New Taipei City, Taiwan; e Institute of Biomedical Sciences, MacKay Medical College, New Taipei City, Taiwan.

**Keywords:** delirium, elderly spouse caregiver, hypercalcemia, vitamin D intoxication

## Abstract

**Rationale:**

Vitamin D deficiency is common among the elderly due to limited sunlight exposure, increasing osteoporosis risk. Over-supplementation poses risks, especially with caregiver involvement.

**Patient concerns:**

Rising vitamin D overdose cases underscore the need for better education on safe intake and monitoring practices to prevent toxicity, particularly hypercalcemia from excessive doses.

**Diagnoses:**

This case report details the clinical presentation and management of a woman who exhibited progressive lower limb weakness, delirium, and abdominal distension over a 3-day period. Initial assessments ruled out intracranial hemorrhage but revealed significant electrolyte imbalances, including hyponatremia, hypokalemia, hypomagnesemia, and severe hypercalcemia. Upon further inquiry, it was discovered that the patient had consumed an excessive amount of liquid Vitamin D3 over the preceding 3 weeks, surpassing the threshold for vitamin D intoxication.

**Interventions:**

Intravenous fluid hydration was initiated to promote calcium excretion. Bisphosphonates and calcitonin were administered to reduce serum calcium levels. Electrolyte imbalances, including hyponatremia, hypokalemia, and hypomagnesemia, were corrected. Continuous monitoring and supportive care were provided in the geriatric ward.

**Outcomes:**

After these treatments, the patient’s electrolyte levels stabilized, and her symptoms, such as confusion and muscle weakness, gradually improved, leading to a full recovery.

**Lessons:**

This case underscores the importance of recognizing hypercalcemia, particularly in the elderly, where symptoms may manifest nonspecifically. Additionally, it highlights the potential risks associated with medication errors and inadvertent overdoses, particularly in situations involving elderly spousal caregivers.

## 1. Introduction

Vitamin D deficiency is a prevalent issue among the elderly due to reduced outdoor activity and limited sunlight exposure, which compromises calcium absorption and increases osteoporosis risk. To address this, vitamin D supplementation at a standard dosage of 800 IU per day is commonly recommended for older adults. However, the potential for over-supplementation poses significant risks, especially when elderly caregivers are involved. Recent data from the American Association of Poison Control Centers’ National Poison Data System show a rising trend in vitamin D overdose cases, particularly among adults over 20. This issue is exacerbated by widespread self-medication, highlighting the need for better education and intervention strategies.

Hypercalcemia, often associated with malignancies or hyperparathyroidism, can also result from excessive vitamin D intake. Recognizing hypervitaminosis D in elderly patients is essential, as high-dose supplementation can easily lead to toxicity. Addressing these challenges requires a multifaceted approach, including vigilant monitoring of supplement intake, education on safe medication practices, and support for elderly caregivers.

## 2. Case report

This case pertains to a 77-year-old woman with a medical history significant for major depressive disorder and hypertension. Approximately 1 month ago, she was discharged from the hospital following an episode of hypertensive urgency. During her hospitalization, laboratory investigations revealed a deficiency in vitamin D levels, with a total 25(OH)-D measurement of 19.70 ng/mL. Consequently, supplementation with vitamin D was recommended for the patient. Patients subsequently self-administered over-the-counter (OTC) vitamin D supplements, potentially exposing themselves to risks of adverse effects on the body. She was subsequently transferred to our emergency department from her psychiatrist’s clinic due to reported confusion and muscle weakness over the past 3 days. According to her husband, she had experienced bilateral hand tremors and weakness in her lower limbs, accompanied by an unsteady gait during this time frame. Additionally, 2 fall episodes occurred the day before yesterday and last night, resulting in a right posterior head injury. Fortunately, there was no loss of consciousness, nor any observable redness, pain, bruising, or hematoma over her trunk and limbs. In the days leading up to her clinic visit, the patient exhibited disoriented speech and engaged in self-talking without signs of drowsiness. In addition, she reported constipation, although she did not present with fever, nausea, vomiting, abdominal pain, decreased urine output, or limb edema.

**Figure 1.**
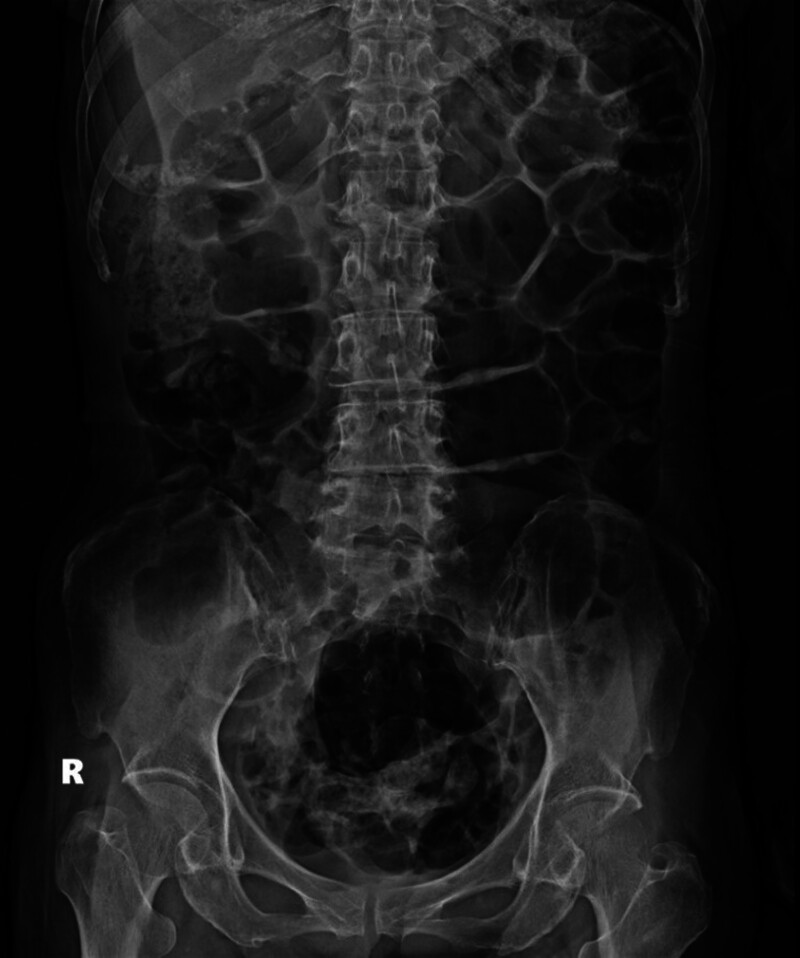
Kidney, ureter, and bladder X-ray of this patient.

**Figure 2.**
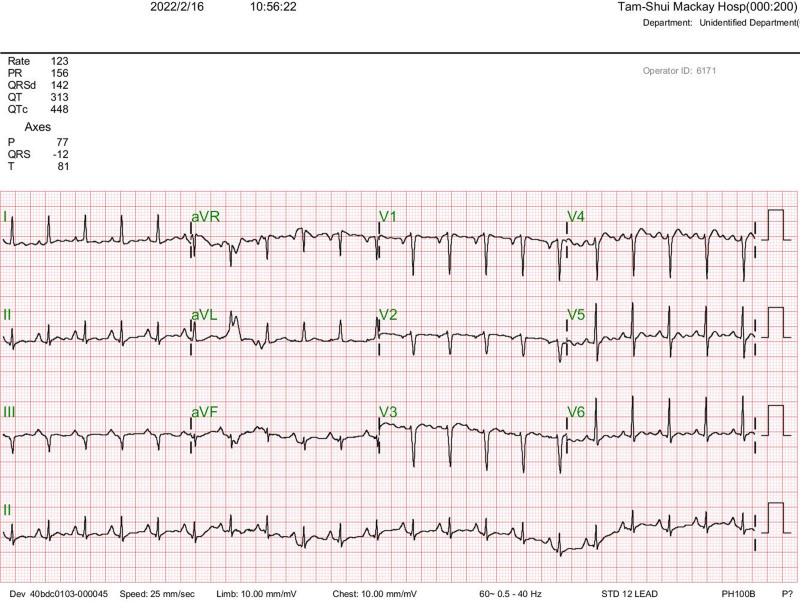
Electrocardiogram of this patient.

**Figure 3.**
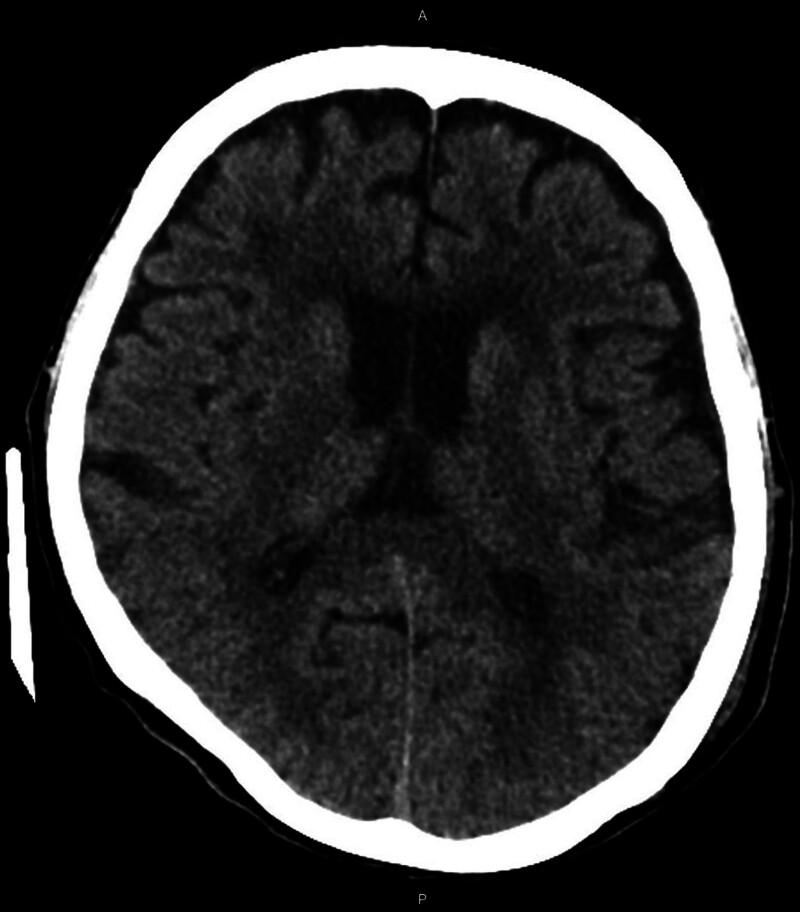
A head computed tomography of this patient.

**Figure 4.**
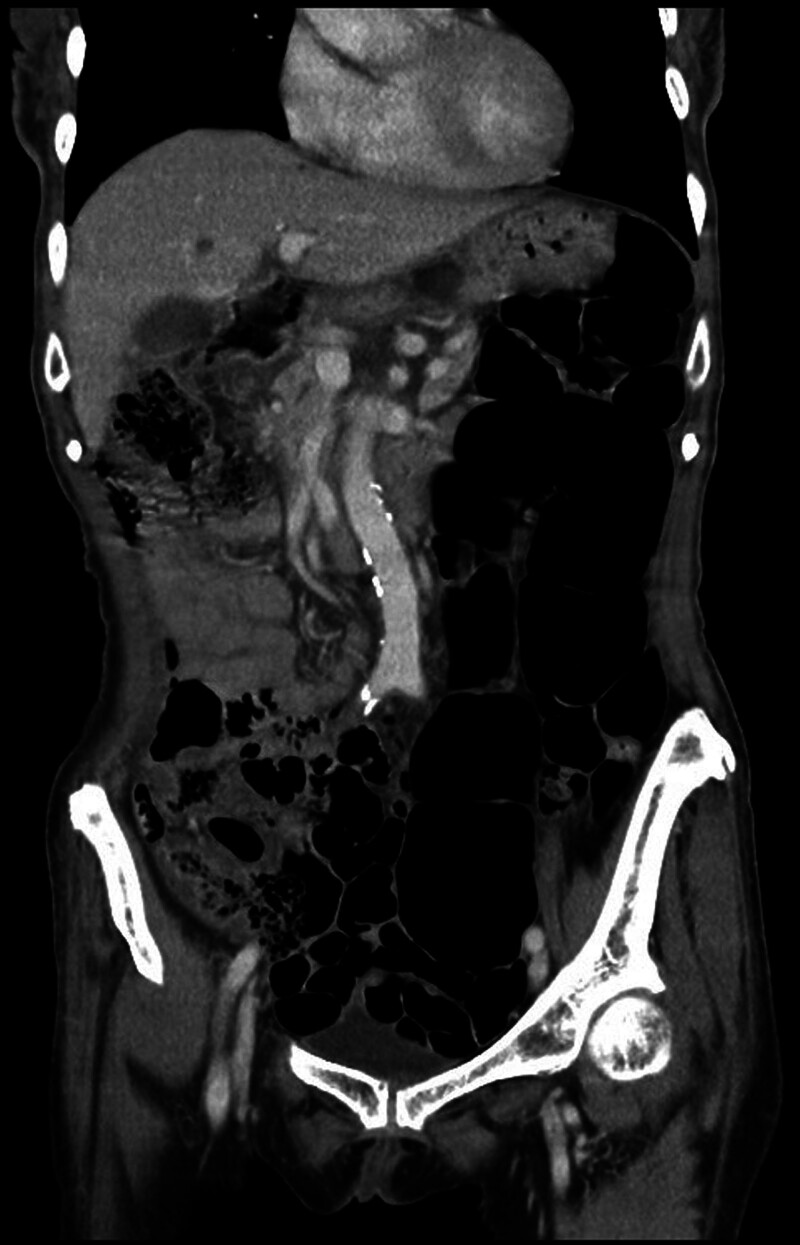
An abdominal computed tomography of this patient.

**Figure 5.**
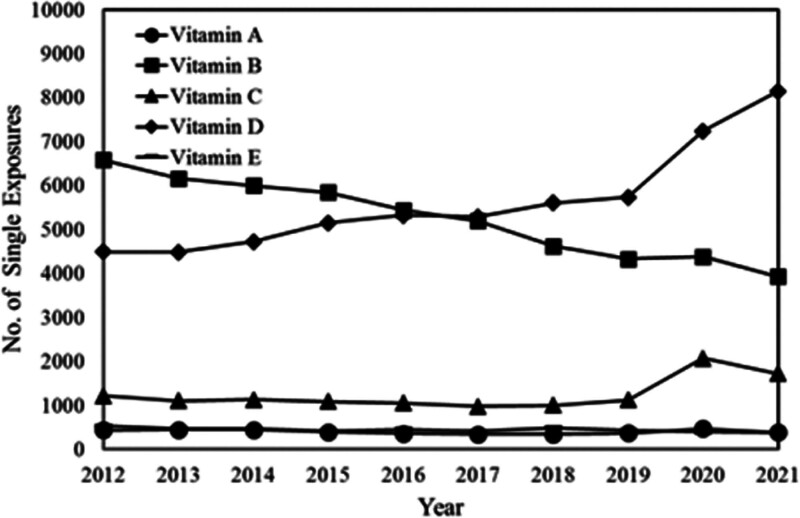
The demographic profile instances of single substance pharmaceutical exposure cases to vitamins A, B, C, D, and E from 2012 to 2021.

**Figure 6.**
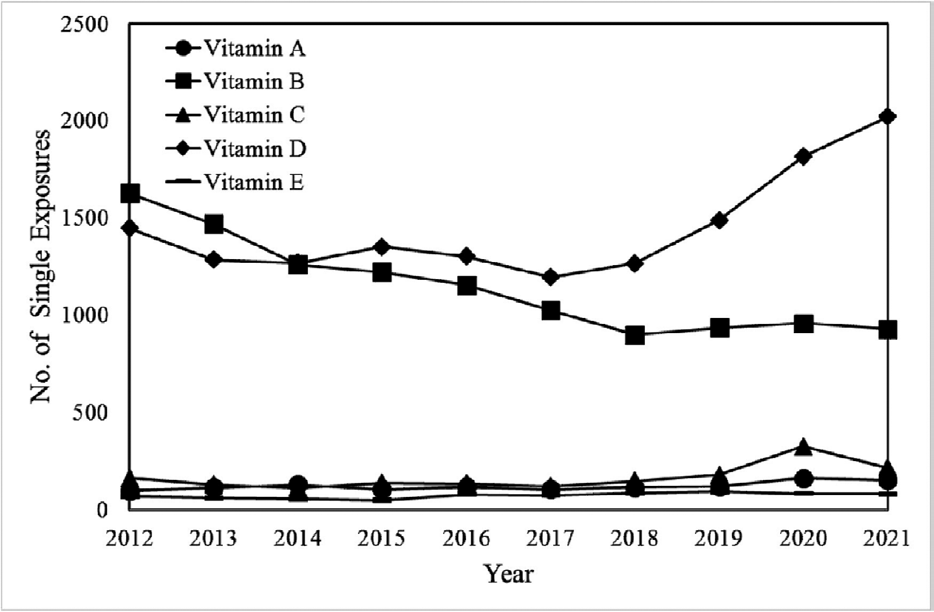
The demographic profiles of single substance pharmaceutical exposure cases to vitamins A, B, C, D, and E in individuals aged 20 and above from 2012 to 2021.

The physical examination revealed a soft abdomen upon palpation, with tympanic percussion noted without any signs of tenderness. In addition, dry skin and mild swelling were observed in the right posterior scalp. Further diagnostic investigations were conducted: Abdominal (kidney, ureter, and bladder) X-ray (Fig. 1) revealed a distended bowel lumen. Electrocardiogram findings did not indicate QTc shortening or arrhythmia (Fig. 2). Brain computed tomography scan did not reveal overt intracranial hemorrhage (Fig. 3). Abdominal computed tomography scan disclosed distended small bowel loops and colon, with feces and air accumulated from the ascending colon to the rectum (Fig. 4). The diagnostic findings from Figures 3 to 6 provide valuable insights that help rule out acute disease problems involving the heart, brain, and bleeding. Although her renal and liver function were within normal range, her blood test showed hyponatremia. Despite her renal and liver function being within normal range, the blood test revealed significant electrolyte imbalances, including hyponatremia (Na: 134.7 mEq/L), hypokalemia (K: 2.8 mEq/L), hypomagnesemia (Mg: 1.69 mg/dL), and severe hypercalcemia (Ca: 14.91 mg/dL). Subsequently, a thorough reevaluation of her history was conducted. It was discovered that the patient’s husband, who is older than the patient herself, had been administering liquid vitamin D3 to her over the past 3 weeks. Shockingly, she had consumed a staggering 18 bottles of liquid vitamin D3 during this time period, with each bottle containing 71,200 IU of vitamin D3, totaling an astonishing intake of 1,281,600 IU. Following the diagnosis of vitamin D toxicity, immediate interventions were initiated to address the electrolyte imbalances and manage the toxicity. Fluid hydration, bisphosphonates, and calcitonin were administered to counteract the hypercalcemia and facilitate calcium excretion. Subsequently, the patient was admitted to the geriatric ward for ongoing monitoring and care. Two days after admission, the level of 25(OH)-D total was rechecked and found to be elevated at 181.00 ng/mL, well above the toxic threshold (>100 ng/mL). Fortunately, the serum parathyroid hormone level was within the normal range. With the correction of electrolyte imbalances and appropriate management of vitamin D toxicity, the patient’s consciousness level and muscle power gradually returned to baseline, indicating a successful resolution of the acute symptoms associated with vitamin D toxicity. Continued monitoring and supportive care were provided to ensure the patient’s continued recovery and well-being.

## 3. Discussion

Hypovitaminosis D is indeed prevalent among the elderly, often stemming from reduced outdoor activities and limited sunlight exposure. This deficiency contributes to osteoporosis by hampering the absorption of calcium from the gut. Consequently, it is estimated that up to one-half of individuals over the age of 65 in North America struggle to maintain optimal bone density.^[1]^ Hence, numerous vitamin D supplements have been advocated to address this widespread deficiency among the elderly. A daily dosage of 800 IU of vitamin D has been commonly recommended for this demographic.^[2]^ Indeed, the potential for over-supplementation arises when caregivers, particularly elderly spouses, encounter challenges in administering the correct dosage or adhering to prescribed frequencies. This scenario underscores the importance of precise medication management, as excessive supplementation can lead to adverse outcomes.

The annual reports from 2012 to 2021 provided by the American Association of Poison Control Centers’ National Poison Data System have highlighted a notable surge in cases involving vitamin D overdose.^[3–12]^ Specifically, the data reveals a 1.8-fold increase in the number of patients experiencing vitamin D overdose across all age groups during this period. When focusing solely on adults over 20, the rise remains significant, albeit slightly lower, at 1.4 times (Figs. 5 and 6). Furthermore, the reports highlight a significant observation: incidents of excessive vitamin D intake outnumber cases involving excessive intake of vitamins A, C, and E by a factor of approximately 10. This discrepancy underscores a specific concern surrounding vitamin D supplementation and its propensity for overdose compared with other vitamins.

Self-medication poses a significant challenge in public health, with its prevalence varying considerably across the globe. It encompasses the use of industrial, traditional, home remedies, and various supplements without professional guidance. The prevalence of self-medication varies widely among different populations and countries. Estimates suggest rates of approximately 22% in Spain, 53% in Mexico, 50% in India, 60% in China, 60% to 90% in Nigeria, and 41% in Iran.^[13]^ The analysis conducted by Rafati et al^[13]^ indicates that the average prevalence of self-medication among the elderly is approximately 36%. This finding underscores the substantial involvement of older individuals in self-medication practices, highlighting the need for targeted interventions and education on safe medication management in this demographic.

In Taiwan, an impressive 84% of individuals actively engage in various self-care practices, indicating a commendable level of health consciousness within the population. However, when confronted with mild symptoms, the majority—64%—choose to seek medical consultation, while only a minority—34%—opt for purchasing OTC medications. This preference underscores the enduring reliance on healthcare professionals for treatment among the Taiwanese populace. Regarding financial commitment, the average monthly expenditure on health-related products for self-care stands at NT 1030. In contrast, spending on OTC medicines for self-treatment averages just NT 275 per month. When considering age demographics, individuals aged 55 to 59 demonstrate the highest level of attention to self-care practices, whether it involves consuming healthy foods or being mindful of dietary nutrition. This highlights a notable trend among middle-aged individuals in prioritizing their health and well-being through proactive self-care measures.^[14]^ Among older adults in Taiwan, dietary supplements are commonly used, with multivitamins and minerals, vitamin E, calcium, and fish oil being among the most popular choices. Data reveal that 32.4% of men and 42.7% of women aged 53 or older reported regularly or occasionally consuming at least 1 type of dietary supplement within the past 12 months. This highlights the widespread use of dietary supplements among older individuals in Taiwan, suggesting a prevalent interest in maintaining health and well-being through supplementation.^[15]^ Based on the survey findings, it is evident that the elderly population has a prevalent habit of taking vitamin supplements. However, it is crucial to consider that the elderly often experience a slower metabolism and are at a higher risk of cross-medication, wherein multiple medications interact with each other. This combination of factors underscores the importance of careful medication management and monitoring among older individuals, particularly regarding the use of vitamin supplements and potential interactions with other medications.

Patients with hypercalcemia may show nonspecific symptoms such as changes in consciousness, weakness, or gastrointestinal issues. It is important to note that over half of these patients have underlying malignancies or hyperparathyroidism.^[16]^ Hypervitaminosis D, leading to hypercalcemia, often results from iatrogenic or accidental overdose. High-dose supplementation, typically recommended for populations at high risk of fractures, such as 1000 IU per day, can be safe. Indeed, as indicated, exceeding 10,000 IU per day may lead to vitamin D intoxication. With the patient consuming a total of 1,281,600 IU over 3 weeks, the calculated average daily intake of approximately 61,000 IU far surpasses this threshold, placing the patient at significant risk of vitamin D intoxication and subsequent complications such as hypercalcemia.^[17]^ This case underscores the potential risks associated with elderly spouse caregivers administering medication and health supplements, highlighting the challenges in ensuring accurate dosing and frequency. Consequently, in patients presenting with delirium, particularly in the geriatric population, meticulous history-taking is crucial. In addition, electrolyte surveys should be expanded to include calcium, magnesium, and phosphate levels to promptly identify and address toxic imbalances. This comprehensive approach is essential for ensuring the safety and well-being of elderly patients, especially in situations where caregiver involvement may pose additional risks.

**Key Points**

- Iatrogenic vitamin D intoxication should be carefully considered in patients presenting with hypercalcemia, particularly among the elderly population.
- Detailed history-taking is essential when assessing elderly patients, especially in cases where medication or supplement use may contribute to adverse effects.
- “Elder Care by Elderly People” poses a significant risk of medication errors and potential overdose, highlighting the importance of vigilant monitoring and support for elderly caregivers to ensure patient safety.

## Author contributions

**Conceptualization:** Ching-Hsiang Yu.

**Writing – original draft:** Ching-Hsiang Yu, En-Chih Liao.

**Data curation:** Hsiang-Kuang Tseng.

**Writing – review & editing:** Hsiang-Kuang Tseng, Ding-Kuo Chien, En-Chih Liao.

**Formal analysis:** Ding-Kuo Chien.

**Resources:** Ding-Kuo Chien, En-Chih Liao.

**Supervision:** En-Chih Liao.
